# Mammoth interatrial septal aneurysm in the ICE age

**DOI:** 10.1186/1476-7120-5-30

**Published:** 2007-09-25

**Authors:** Ravinay Bhindi, John Timperley, Oliver J Ormerod

**Affiliations:** 1 Cardiology Department, John Radcliffe Hospital, Headley Way, Headington, Oxfordshire, OX3 9DU, UK; 2 Department of Cardiovascular Medicine, University of Oxford, John Radcliffe Hospital, Headley Way, Headington, Oxfordshire, OX3 9DU, UK

## Abstract

**Background:**

Intracardiac echocardiography (ICE) is a useful imaging modality that is now being used more widely to assist in the percutaneous closure of atrial septal defects (ASD) and patent foramen ovales (PFO).

**Case presentation:**

A 42 year old lady with a history of transient ischaemic attacks and migraine underwent percutaneous closure of an ASD. Intraprocedural ICE demonstrated a mammoth billowing multiperforated interatrial septal aneurysm in association with a secondum ASD.

**Conclusion:**

ICE provides excellent adjuvant imaging during percutaneous closure of intracardiac shunts, in this case demonstrating a 'mammoth' interatrial septal aneurysm.

## Background

Intracardiac echocardiography (ICE) is a useful imaging modality that is now being used more widely to assist in the percutaneous closure of atrial septal defects (ASD) and patent foramen ovales (PFO).

## Case presentation

**Figure 1:**
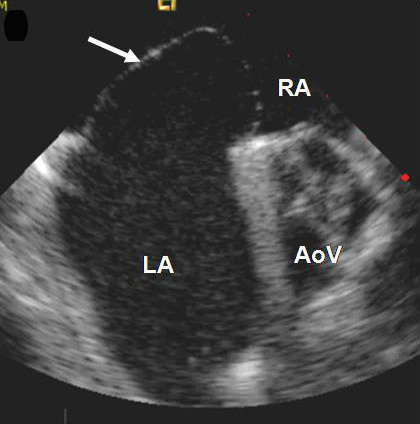
Image obtained using intracardiac echocardiography (ICE): Demonstrating a large interatrial aneurysm (arrow) billowing into the right atrium (RA) with an excursion of over 22 mm.

**Figure 2:**
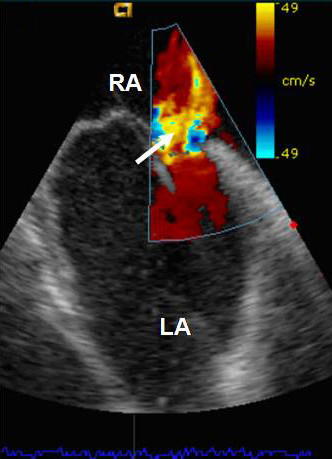
Image obtained using intracardiac echocardiography (ICE): With colour flow mapping (arrow) of the interatrial septum demonstrating a large ASD with shunting.

**Figure 3:**
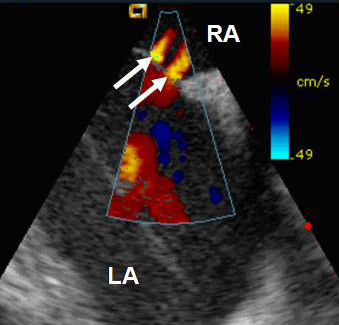
Image obtained using intracardiac echocardiography (ICE): With colour flow mapping of the interatrial septum demonstrating 2 jets (arrows) across the septum.

A 42 year old lady with a history of previous transient ischemic attacks and migraines was found to have a secundum ASD and an aneurysmal interatrial septum on contrast transthoracic echocardiography. She then went on to undergo percutaneous closure of the ASD, during which ICE demonstrated a dramatic giant multiperforated aneurysmal interatrial septum with a secundum ASD on Doppler flow imaging (Figures 1, 2, 3).

## Discussion

An aneurysmal interatrial septum is thought to be the harbinger of potential embolic thrombus and its presence increases the risk of a stroke significantly particularly in association with a PFO and is thus of clinical importance [1]. Adjuvant ICE imaging has permitted such cases to be performed as a same day discharge procedure without the need for general anaesthesia and provides detailed imaging to enable rapid, accurate closure of intracardiac lesions.

## Conclusion

This case demonstrates a "mammoth" billowing multiperforated interatrial septal aneurysm in association with a secondum ASD and illustrated elegantly by ICE.

## Competing interests

The author(s) declare that they have no competing interests.

## Authors' contributions

All authors contributed to the preparation of this manuscript.
